# Vertical Fiberglass Micropiles as Soil-Reinforcing Elements

**DOI:** 10.3390/ma15072592

**Published:** 2022-04-01

**Authors:** Mohanad Muayad Sabri Sabri, Nikolai Ivanovich Vatin, Renat Rustamovich Nurmukhametov, Andrey Budimirovich Ponomarev, Mikhail Mikhailovich Galushko

**Affiliations:** 1 Peter the Great St. Petersburg Polytechnic University, 195251 Saint Petersburg, Russia; vatin@mail.ru (N.I.V.); nrenatkazan@gmail.com (R.R.N.); andreypab@mail.ru (A.B.P.); 2 Saint Petersburg State University, 190999 Saint Petersburg, Russia; mig94@mail.ru

**Keywords:** fiberglass, soil reinforcement, vertical reinforcement, FRP pile, screw micropile, fiber-reinforced composite, deformation

## Abstract

This article is dedicated to developing a ground improvement technique using vertically oriented reinforcement elements prefabricated utilizing fiberglass pultruded pipe and helical shape wideners at the bottom toe. Structures of the prefabricated helical micropiles varied by the length and cross-section area introduced into the soil massive as reinforcing bearing elements. The effect of the reinforcements geometry variation was investigated through a reinforcement factor (*µ*), based on which a calculation method for measuring settlement of reinforced soil has been previously developed Full-scale field plate load tests were performed before and after reinforcing the soil to investigate the changes in the soil stiffness after the reinforcement process. Comparative analysis between the reinforced and reference soft sandy soil indicates an average increase in the deformation properties of the fiber reinforced soils by 8%, 30%, 63% at the applied pressures of 100, 300, and 550 kPa, respectively. The influence of the fiber reinforced polymers (FRP) geometrical properties on the final composite settlement was determined. A comparative analysis of the calculated and the actual plate load tests results reveals that the previously proposed settlement calculation method is adequate for further development.

## 1. Introduction

The settlement of buildings and structures due to soft soils conditions is a permanent problem in geotechnical practices [1,2]. The scope of the problem widens when dealing with foundations built on slope grounds, soft soils, areas located under seismic impacts, and other weak geological circumstances [3]. The traditional ground improvement materials have many restrictions, are costly and not feasible, and, in many cases, might lead to electrochemical corrosions, threatening the whole overlaying constructions. Thus, developing practical and effective methods is a current task to provide stable, safe, and economic reconstruction [4].

Thus far, different reinforcement materials have been developed and implemented utilizing various inclusion in the soil’s massive mechanisms, including environmentally friendly geopolymers, expanding resins, natural materials such as bamboo, fiberglass materials, etc. [5,6,7,8].

One of the most common and effective ground improvement techniques used in practical applications is the change in the load operating conditions on the ground using reinforcing elements in a term of micro and helical piles. In this regard, several techniques have been developed, including the horizontal micro piles [9]. Soil reinforcement can be widely applied in large projects such as roads, embankments, etc., and it can be applied to long piles with deep soft sublayers in these types of projects.

Lv and Liu performed a comparative analysis for circular shape vertical reinforcement and X-shape cast in-situ piles [10]. Several investigations of horizontal reinforcement behavior were undertaken by Naggar [11,12,13]. Soft soils’ stiffness increase by horizontal reinforcement was proved by Ponomarev [14,15]. Usmanov analyzed the method of weak soils reinforcement using layer by layer compaction and high strength geosynthetic materials (HSGM) reinforcement [16]. One of the particular options of sand or gravel type reinforcing piles application is geosynthetic mats bounding the shape of bored piles. Shenkman [17] and Maltseva [18] proposed a settlement calculation method after the reinforcement process. Natural foundation underpinnings were studied by Waruwu [19].

Popov proposed a calculation method considering the stiffness of vertically installed soil-reinforcing elements [20]. Sabri [5,6] investigated soil reinforcement behavior using an expandable polyurethane resin injected into the soil mass in the hydrofracturing mode. In further studies, Sabri [7] developed a new method to calculate the reinforced deformation and strength properties of expandable polyurethane resin soils after the injection process, considering the resin as solid reinforcing elements in the vertical directions.

It is important to note that vertical reinforcement is specified in the codes presented by traditional materials such as concrete, geosynthetic-encased column, steel, and crushed stone. However, fiberglass or similar composite materials are not detailed in the standards, according to Shalenny [21].

The soil vertical reinforcement is one of the existing techniques to improve ground strength and reduce foundations settlements. The term reinforcement stands for the strengthening of the soil by penetration into the soil mass of additional elements with higher strength properties and different shapes and materials. Cement grouting and vertical expanding geopolymers as a rigid inclusion have been studied by [1,2,5,6,7,22,23,24,25,26,27,28,29]. A search for materials to be used for the soil’s strengthening is one of the most perspective directions from a sustainable engineering point of view since recycled materials can be applied [30].

**Figure 1:**
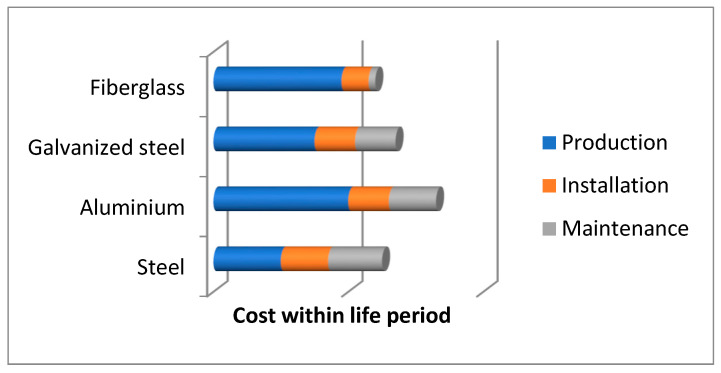
Materials’ comparison [36].

In recent years, the use of fiberglass in various directions, including FRP in geotechnical applications, has arisen for many reasons. According to Krishan [31], the main benefits of FRP could be sorted as follows:

- The material does not require rust protection, and it leads to a reduction in expenses for maintenance;
- Maintenance is cost-effective, as described by Pando [32]. Fiberglass materials do not require annual coating or painting as steel needs. However, the cost-effectiveness of this approach is not achieved immediately, as shown in Figure 1;
- The high strength and low density of fiberglass, $R_{bend}$ is equal to 690 MPa, and ρ is equal to 1600 kg/m^3^, providing evidence of the future trend of FRP application growth;
- Low density leads to cost reduction in transportation. Boyarintsev investigates the application of fiberglass in permafrost areas [33];
- Less labor and light equipment are required for implementation and ease of installation to the ground;
- A relatively high thermal conductivity factor of 0.3–0.6 W/K·m;
- Fiberglass does not allow electric contact with the ground. This benefit can be used for lightweight thin-walled steel profiles researched by Nazmeeva and Garifullin [34,35] since the foundation’s electric corrosion influence is eliminated;
- Mohajerani [8] studied the life cycle of fiberglass piles exposed to severe conditions produced by Bedford technology. Compared with wooden piles, it was proved that fiberglass piles become cheaper after six years in service, considering the maintenance cost;
- No additional weight is added to the soil layers beneath the reinforcement due to the FRP low weight.

Various numerical and experimental studies have been conducted to investigate micropiles used for soil reinforcement in geotechnical applications [37,38,39,40,41,42,43,44,45].

The behavior of screw steel vertical micropiles was studied by Papadopoulou [46]. The geometry of assessed micropiles is similar to the investigated in this article reinforcing elements. Pando [32] and Valez [47] tested and renovated the structure of composite piles. Valez [47] also investigated the behavior of carbon fiber piles in stabilized and non-stabilized soft soils besides fiberglass piles. The results of this study proved higher adhesion and bearing strength, compared with steel piles. Sirimanna [48] tested epoxy coating to resolve an issue of delamination. The epoxy coating was injected between shell and core, improving cohesion and dynamic load transfer for the driven piles. Additional investigations are required to decrease delamination influence on the piles’ bearing strength, especially for lateral and torsional forces.

Existing studies on micropiles as soil-reinforcing elements are insufficient to understand the investigated composite structures and their combined behavior within soft soils [31]. Shashkin and Ulitskii [49] noted that most of the existing methods applied for settlements calculation have low correlations with the actual test results observed during intensive experimental studies.

A settlement calculation method for soil reinforced by fiberglass micropiles in the vertical directions was proposed by Nurmukhametov [50].

The literature analysis has shown that fiberglass materials have many advantages in practical applications. Fiberglass micropiles are easy to install, cost-effective, and have high load bearing strength in horizontal and vertical directions. Therefore, recent years have established new trends to use FRP as vertical soil reinforcement. However, FRP applications are limited due to the lack of theoretical and practical justifications that clarify the ground improvement trends using these materials and the absence of calculation methods of the deformation and strength properties after the reinforcement process. Additionally, connection details of fiberglass materials with steel and concrete are not well developed and studied yet.

This article aims to develop a soil reinforcement method using fiberglass micropiles installed into the ground in the vertical directions and determine the changes in the ground deformation behavior after its reinforcement by FRP micropiles using full-scale field plate load tests. Different lengths and cross-section areas of fiberglass micropiles were used in this study to investigate the influence of the FRP micropiles’ geometrical parameters on the deformation behavior after the reinforcement. A comparative analysis of the experimental and theoretical results was carried out as a verification process. The theoretical settlement was calculated based on the author’s previously proposed calculation method [50]. 

The main objectives of this study are as follows:

- To develop a soil reinforcement method using fiberglass micropiles installed into the ground vertically;
- To determine the ground deformation behavior changes after its reinforcement by FRP micropiles using full-scale field plate load tests;
- To assess the influence of FRP’s length and cross-section area on the final settlement of the reinforced ground;
- To investigate the influence of the applied load on the settlements of soils reinforced by FRP elements;
- To justify the settlement calculation method through number of in-field plate loading tests. Method was previously proposed by one of the authors of this article [50].

## 2. Materials and Methods

### 2.1. The Investigated FRP Micropiles

Four micropiles samples were inserted into the soil mass as reinforcing elements in the vertical directions. The investigated micropiles are differentiated by their diameter, materials, and screw numbers. The materials used to prefabricate the FRP are produced by “Composite Group LLC, Balashikha city, Russia”.

The first micropile was made of fiberglass with a diameter of 100 mm. The second and the third micropile samples were fiberglass, with their diameters reduced to 75 mm. However, the number of screws differentiated these two samples. One sample had a screw at the bottom toe, and one sample had two screws at the bottom toe and in the middle. The fourth sample was made from steel pipe with a diameter of 100 mm.

**Figure 2:**
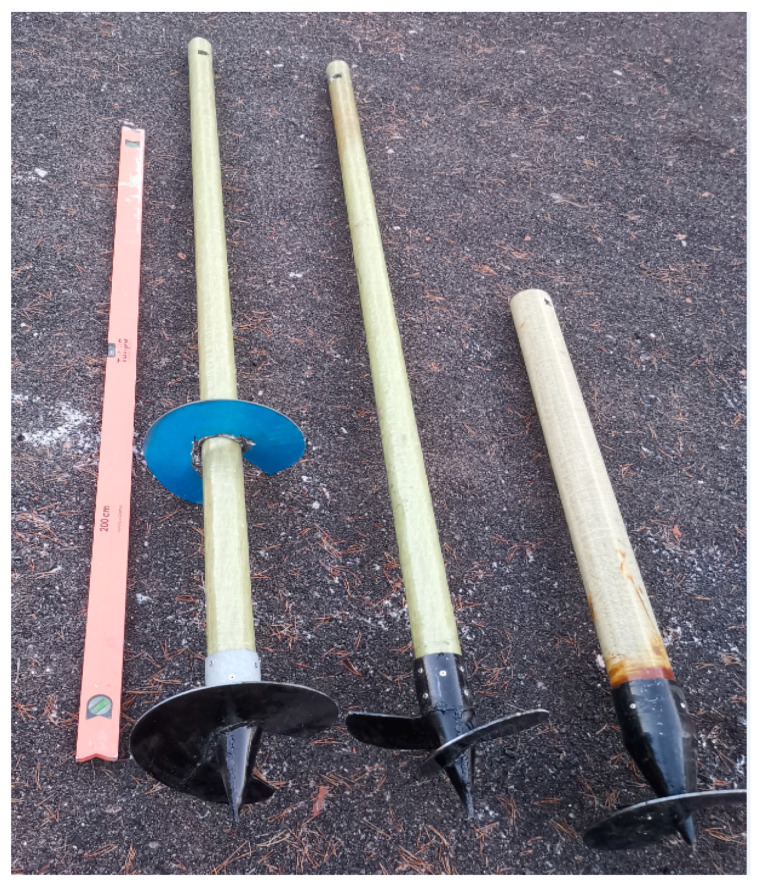
The investigated fiberglass reinforcing elements.

Moreover, the micropiles were discerned by their lengths. The length of the first and fourth micropiles, the diameter of 100 mm, was 80 cm, while 200 cm were chosen for samples, the diameter of which was 75 mm. Figure 2 shows the investigated fiberglass micropiles.

The reinforcing elements had steel screws with diameters of 300 mm. Screws were fixed to the fiberglass pipes by stainless rivets. The fiberglass pipes were fabricated using a shear bending roving to increase the shear connection between the soil and vertical reinforcing elements. In addition, the steel screws at the bottom toe act as additional elements that enhance the load distribution on the composite system (soil-reinforcing elements). The geometrical and material properties of the investigated micropiles are shown in Table 1.

The geometry of the investigated FRP micropiles was similar to the screw steel vertical micropiles that were investigated by Papadopoulou [46].

In addition, *µ* is a reinforcement area factor calculated as per formulas previously proposed by the authors [50], which is defined as

(1) $$\mu =\frac{\gamma_{frn}A_{aef}+\gamma_{Rrn}A_{aeR}}{A_{rp}},$$

where *γ_frn_* and *γ_Rrn_* are the factors of the fiberglass reinforcement’s cross sections and screws’ areas, *A_aef_* is the area of FRP elements’ friction surface, and *A_aeR_* is the area of reinforcing elements’ screw imitated by the washer fixed to the bottom toe.

### 2.2. In Situ-Plate Load Test

Full-scale plate load tests were performed to investigate the load–settlement relationship before and after the soil reinforcement by fiberglass micropiles in the vertical direction. The investigated reference soil was uncompacted soft sand.

**Figure 3:**
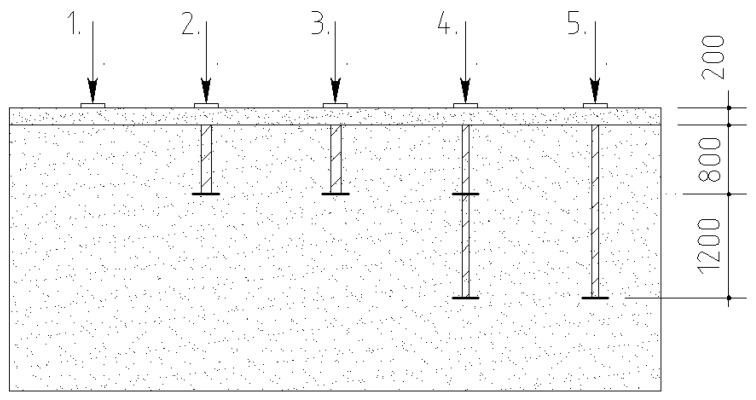
Scheme of the tested samples: (1) reference soil; (2–5) FRP-reinforced soil.

**Figure 4:**
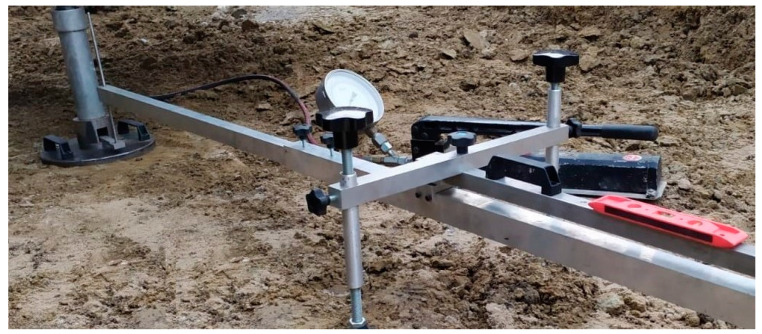
The in-situ plate load test system used in the experiment.

The load was applied using concrete slabs, with a total weight of 12 tons/m^2^ (118 kPa). A hydraulic jack was used to transfer the load to the steel plate through supported steel beams. The load was transferred to the soil mass through the steel plate, the area of which was 600 cm^2^. The samples were loaded through incremental loading stages of 10 kPa. The conditional stabilization time during the deformation process was considered. The allocation scheme of the micropiles and the reference soil during the testing process is shown in Figure 3. The in-situ plate load test installation is shown in Figure 4.

The obtained results were compared relatively before and after the reinforcement process to determine the changes in the composite deformation properties.

### 2.3. Background on a Previously Proposed Theoretical Settlement Calculation Method

**Figure 5:**
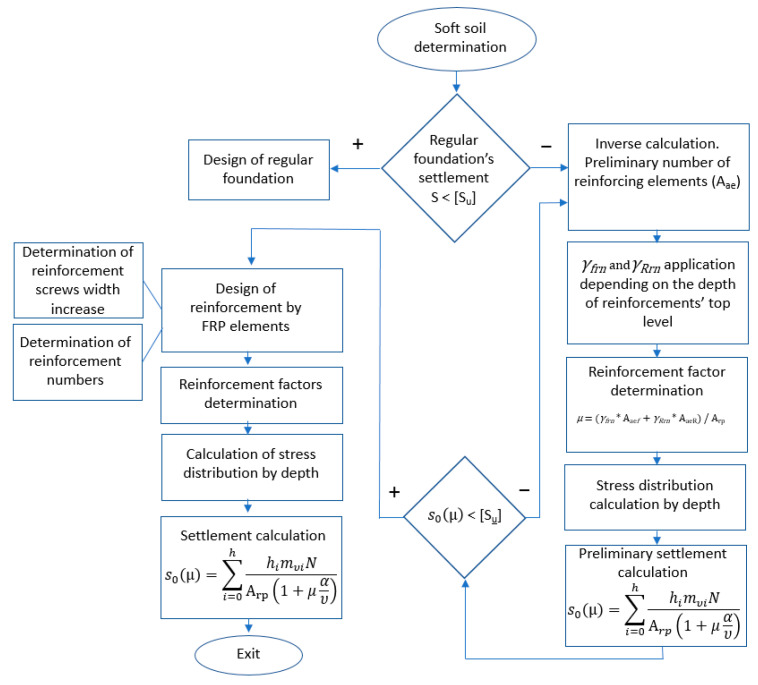
The settlement modeling algorithm previously proposed by [50] for soils reinforced by vertical fiberglass micro-piles.

Furthermore, the settlement of each in-situ tested sample was calculated according to a settlement calculation method previously proposed by one of the authors [50] to verify the reliability of the proposed calculation method (Figure 5).

This previously proposed method assumes the following:

- The soil and the reinforcement form a composite system, in which the reinforcing elements act as bearing elements. The reinforcement and the soil are settled together during the loading process [51];
- Most of the load is transferred to the stiffer member of the composite micropile;
- Stress is distributed between reinforcing elements and weak soil [52];

The proposed calculation method was developed based on the existing studies considering the following:

- The modeling process reflects the deformation process as a linear elastic half-space (linear deformation part). The plastic behavior and failure are not considered in this study, same as they were not considered in the previously proposed calculation method.

## 3. Results and Discussion

### 3.1. Result of the Field Investigations

**Figure 6:**
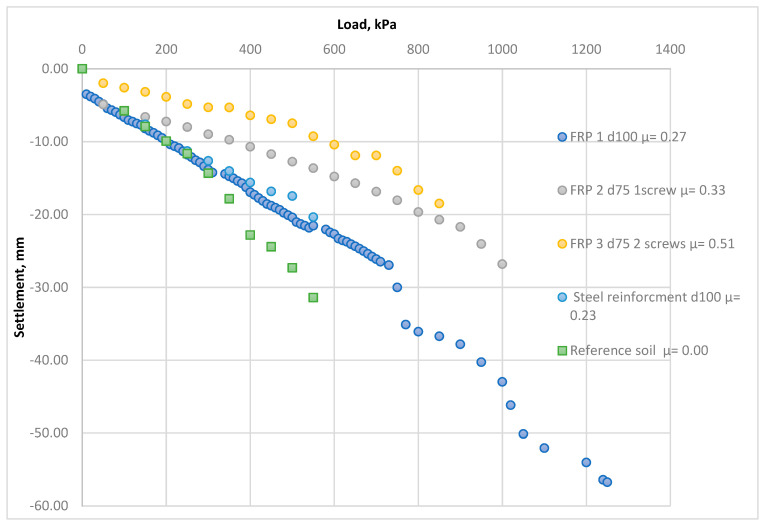
Load−settlement graphs of the tested samples before and after the reinforcement according to the field plate load tests (PLT) results.

Figure 6 shows the in-situ load–settlement relationships before and after the soil reinforcement by various types of vertical FRP micropiles.

Based on a comparative analysis of the results (Figure 6), the following observations were made:

- An increase in the soil deformation modulus was accompanied by a decrease in the soil settlements after soil reinforcement by vertical FRP micropiles was achieved. The average increase in the FRP-reinforced soils of all the investigated samples were 8%, 30%, 63% at the applied pressures of 100, 300, and 550 kPa, respectively;
- The reinforcement area factor *µ* had a direct influence on the ground’s improved properties. Table 2 shows the effect of the *µ* factor variations on the settlement improvements after the reinforcement process;
- The length of the investigated FRP micropiles directly influenced the ground’s improved properties. Reinforced soil by micropiles, whose length equals 2 m, was stiffer than 0.8 m micropiles. The average stiffness increase along with the soil layer depth was 46%;
- Installation of a second screw to the FRP micropiles improved reinforcement performance. The best settlement reduction values were gained using the double screw FRP micropiles, which was justified by increasing the cohesion between the soil and the reinforcing elements. The average settlement reduction using the double screw FRP micropile along the soil layer depth was 63%, compared with a similar geometry one screw FRP micropile;
- The investigated composite’s settlement reduction was enhanced, along with the soil layer’s depth and increased applied load.

**Figure 7:**
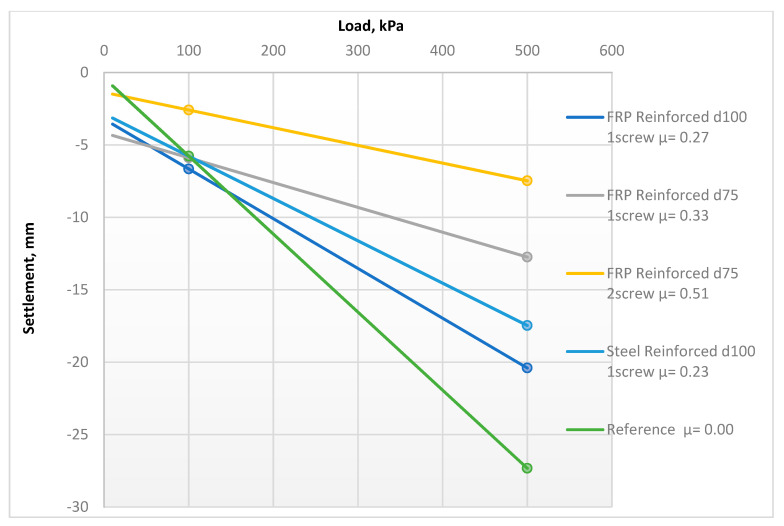
The in situ PLT elastic deformation before and after the reinforcement for varied reinforcement areas.

**Figure 8:**
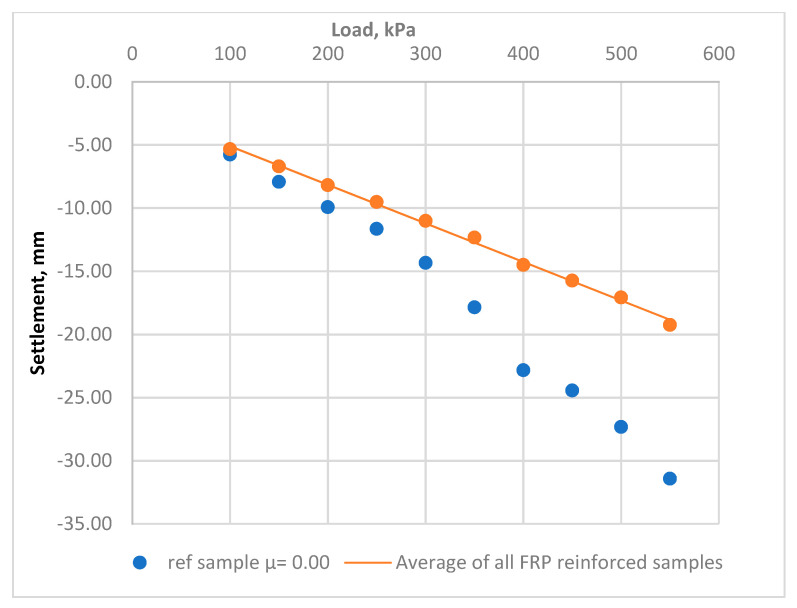
The average elastic load−settlement relationship for all FRP-reinforced soil compared to the reference soil.

The factor used to assess soils’ settlements behavior was the calculated deformation modulus within a stress range of 100–500 kPa. Figure 7 and Table 2 present changes in the deformation properties before and after the reinforcement process. Figure 8 presents the averaged elastic load–settlement relationship for all FRP-reinforced soils, compared with the reference soil.

### 3.2. The Results of the Theoretical Calculations

Furthermore, the settlement’s theoretical calculations for all the investigated samples were determined according to the calculation method previously proposed by [50] (Equations (1)–(5)).

(2) $$s_{0}(\mu )=\sum_{i=0}^{h}\frac{h_{i}m_{\upsilon i}N}{A_{rp}(1+\mu \frac{\alpha }{\upsilon })},$$

where $\alpha =\frac{E_{ae}}{E_{rp}}$ is a ratio of the reinforcing element’s elastic modulus to the soil deformation modulus, *h_i_* is the height of the soil layer, *A_rp_* is an area of soil’s element, *N* is a vertical force applied to the reinforced soil mass, *v* is an elastic–plastic settlement ratio, which is calculated as follows:

(3) $$\nu =\frac{\varepsilon_{e}}{\varepsilon_{e}+\varepsilon_{pl}(t,\sigma_{rp}/R_{rp})},$$

Reference settlement factor $m_{\upsilon i}$ depends on the deformation modulus *E_v_* that is taken from the lab tests by using the following modified equation:

(4) $$E_{\upsilon }=\beta l\frac{\Delta \sigma }{\Delta s’},$$

Factor *β* is developed based on Hook’s law boundary conditions. The current factor depends on a Poisson ratio.

(5) $$\beta =1-\frac{2\upsilon^{2}}{1-\upsilon },$$

**Figure 9:**
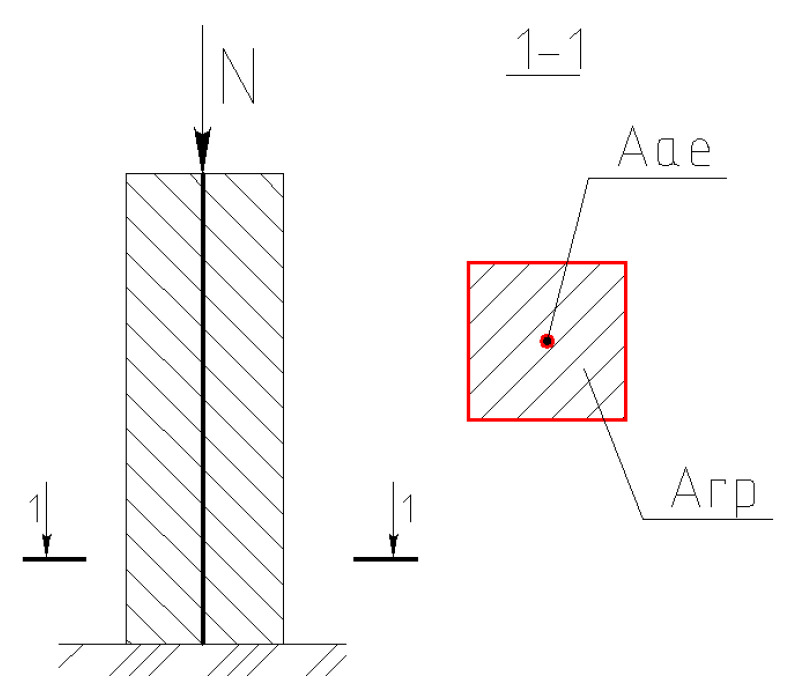
Soil-reinforcing element model. *A_rp_* is the cross-section area of the soil’s element, and *A_ae_* is the cross-section area of one vertical reinforcing element.

Preliminary theoretical and numerical studies were conducted by considering that the reinforced soil mass is a combined system that consists of both soil and reinforcing elements. Figure 9 presents the soil-reinforcing element model. The load was distributed mainly through the reinforcing elements at the first stage due to their higher stiffness. In the next step, reinforcing elements penetrated soft soil layers since they were not supported by stiffer soil beneath. Hence, part of soft soils and reinforcing elements were shifted down together, acting as a composite system in which the reinforcement act as a bearing element. The load was distributed into the soil mass. This analysis approach is similar to the model of soil reinforced by an expandable polyurethane resin developed by Sabri [12].

Finally, the applied load was distributed on the FRP-reinforced soil as a composite orthotropic system (Equation (6)).

(6) $$\varepsilon_{ae}=\varepsilon_{rp}=\frac{\sigma_{rp}}{E^{\prime }{}_{rp}},$$

In addition to the above, model simplification was considered for the application of sensitivity analysis. Quantification of input variables uncertainties was utilized due to differentiated parameters of the same soil within each soil prism and underlayer. It is reasonable to claim that model simplification led to a reduction in the qualitative and quantitative outputs of the proposed calculation method.

**Figure 10:**
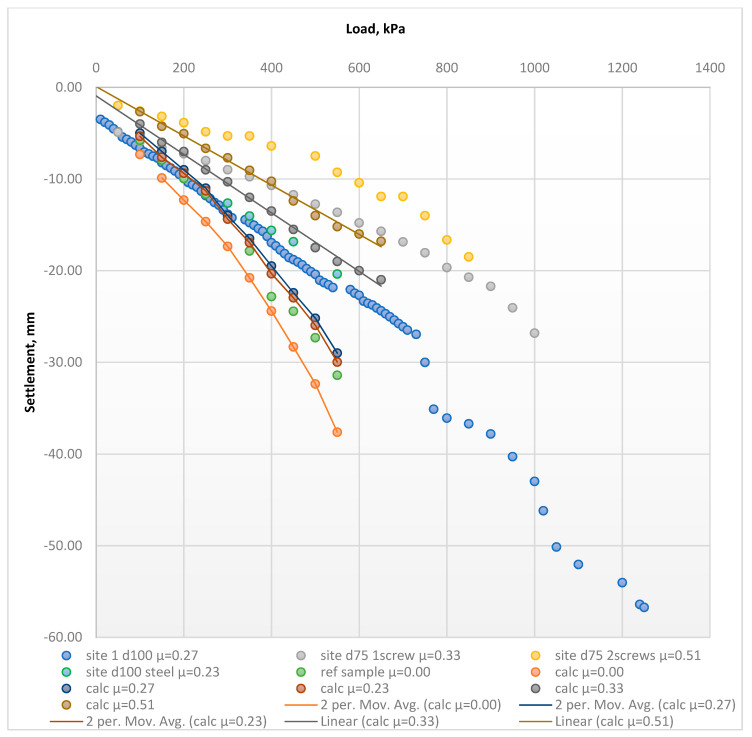
The load−settlement curve of each of the tested in situ samples, as compared with the theoretical calculation results.

**Figure 11:**
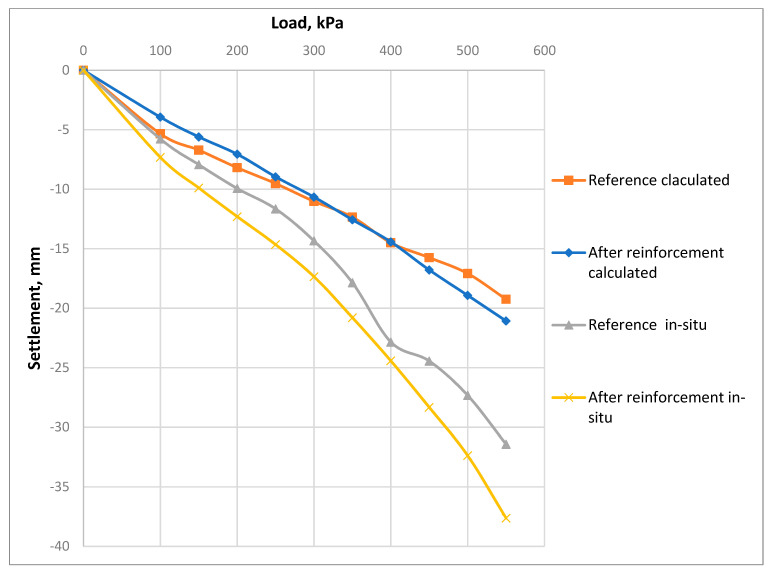
The averaged load−settlement curve of all investigated in situ samples, compared with the averaged theoretical calculation results before and after the reinforcement process.

Figure 10 shows the theoretical calculated and in-situ PLT load–settlement curves before and after the reinforcement process. The averaged load–settlement curve of all investigated in-situ samples, compared with the averaged theoretical calculation results before and after the reinforcement process, are shown in Figure 11.

In the main parametric study, load–settlement curves were considered to be built based on the following initial key factors:

- Current studies that evaluate elastic deformations;
- The applied method was considered for the improvement of weak soils that have *E* < 7 MPa;
- The applied load direction of the current study was limited by the application of vertical pressure;
- The boundary of investigated soil element was limited to the three diameters of a single reinforcing element or to the half distance between two vertical elements but not more than the three diameters of the reinforcing element;
- The model of each reinforcing element was assumed as a vertically oriented linear rod.

A comparative analysis before and after the reinforcement process shows that the theoretically calculated load–settlement relationships reflect the in situ PLT results with sufficient accuracy. It indicates that the previously proposed calculation method is reliable for further development, and it confirms the experimental results, as shown in Figure 10 and Figure 11. Although some discrepancies exist between the theoretical calculation and the experiment results, the obtained results have shown an attempt to approximate the load–settlement curves within the elastic behavior of the soil before and after the reinforcement. Further large-scale studies are still required to investigate the changes patterns in the FRP-reinforced soil’s deformation modulus at different soil depths and applied loads.

## 4. Conclusions

A full-scale field experiment was conducted to develop a soil reinforcement method using vertical fiberglass micropiles and determine the changes in the ground deformation behavior after its reinforcement by various geometrical parameters of FRP micropiles in the reinforcement process. The results of in situ plate load tests were compared with a calculation method previously proposed by one of the authors calculation method, leading to the following outcomes:

1. The soil improvement using fiberglass micropiles as a reinforcing element in the vertical direction was established and proved;
2. The in situ test results enhanced by the theoretical calculation method showed an increase in the deformation properties, accompanied by a decrease in the soil settlements after the soil reinforcement by vertical fiberglass micropiles reinforcing elements. The average increase in the deformation properties of the FRP-reinforced soils was 8%, 30%, 63% at the applied pressures of 100, 300, and 550 kPa, respectively;
3. The reinforcement area factor *µ* had a direct influence on the ground’s improved properties. Table 2 shows the effect of the *µ* factor variations on the settlement improvements after the reinforcement process;
4. The length of the investigated FRP micropiles directly influenced the ground’s improved properties. Soil reinforced by micropiles with a length of 2 m was stiffer than 0.8 m micropiles. The average increase along with the soil layer depth was 46%;
5. Installation of a second screw to the FRP micropiles improved reinforcement performance, which was justified by increasing the cohesion between the soil and the reinforcing elements. The average settlement reduction using the double screw FRP micropile along the soil layer depth was 63%, compared with a similar geometry of one screw FRP micropile.
6. The investigated composite’s settlement reduction was enhanced with the soil layer’s depth and the applied load’s increase. Further large-scale studies are still required to investigate the changes patterns in the FRP-reinforced soil’s deformation modulus at different soil depths and applied loads.

A comparative analysis before and after the reinforcement process shows that the theoretically calculated load–settlement relationships reflect with sufficient accuracy the in-situ results. It indicates that the settlement calculation method previously proposed by one of the authors is reliable for further development.

## 5. Recommendations

Regardless of the achieved results in this field thus far, the following topics still need to be researched:

1. To investigate the behaviour of various soil types reinforced by the proposed FRP micropiles.
2. To develop a generalized model to calculate the FRP-reinforced soils’ settlement and bearing capacity.
3. To investigate the changes patterns in the FRP-reinforced soil’s deformation modulus at different soil depths.
4. An investigation of the long-term durability of this material is required. A more detailed study of fiber glass potential influence on the environment is preferable.

## Figures and Tables

**Table 1:** The geometrical and material properties of the investigated micropiles.

Samples No.	The Material Used	Diameter, mm	Length, mm	Screw, pcs	Reinforcement Factor *µ*
Sample 1	FRP	100	800	1	0.27
Sample 2	FRP	75	2000	1	0.33
Sample 3	FRP	75	2000	2	0.51
Sample 4	Steel	100	800	1	0.23

**Table 2:** Summary of the in-situ deformation modulus before and after the reinforcement.

Location	Before Reinforcement	After Reinforcement			
Reinforcement factor, *µ*	0	0.23	0.27	0.33	0.51
Deformation modulus, MPa	3.69	5.79	6.8	11.59	16.27
